# Farm-Level Risk Factors Associated With Avian Influenza A (H5) and A (H9) Flock-Level Seroprevalence on Commercial Broiler and Layer Chicken Farms in Bangladesh

**DOI:** 10.3389/fvets.2022.893721

**Published:** 2022-06-16

**Authors:** Suman Das Gupta, Guillaume Fournié, Md. Ahasanul Hoque, Joerg Henning

**Affiliations:** ^1^School of Veterinary Science, The University of Queensland, Gatton, QLD, Australia; ^2^Department of Pathobiology and Population Sciences, Royal Veterinary College, University of London, London, United Kingdom; ^3^Department of Medicine and Surgery, Chattogram Veterinary and Animal Sciences University, Chattogram, Bangladesh

**Keywords:** avian influenza, broiler, layer, commercial chicken, H5, H9, risk factor, seroprevalence

## Abstract

A cross-sectional study was conducted to identify farm-level risk factors associated with avian influenza A H5 and H9 virus exposure on commercial chicken farms in Bangladesh. For broiler farms, both H5 and H9 seropositivity were associated with visits by workers from other commercial chicken farms [odds ratio (OR) for H5 = 15.1, 95% confidence interval (CI): 2.8–80.8; OR for H9 = 50.1, 95% CI: 4.5–552.7], H5 seropositivity was associated with access of backyard ducks (OR = 21.5, 95% CI: 2.3–201.1), and H9 seropositivity with a number of farm employees (OR = 9.4, 95% CI: 1.1–80.6). On layer farms, both H5 and H9 seropositivity were associated with presence of stray dogs (OR for H5 = 3.1, 95% CI: 1.1–9.1; OR for H9 = 4.0, 95% CI: 1.1–15.3), H5 seropositivity with hatcheries supplying chicks (OR = 0.0, 95% CI: 0.0–0.3), vehicles entering farms (OR = 5.8, 95% CI: 1.5–22.4), number of farm employees (OR = 5.8, 95% CI: 1.2–28.2), and burying of dead birds near farms (OR = 4.6, 95% CI: 1.2–17.3); H9 seropositivity with traders supplying feed (OR = 5.9, 95% CI: 1.0–33.9), visits conducted of other commercial poultry farms (OR = 4.7, 95% CI: 1.1–20.6), number of spent layers sold (OR = 24.0, 95% CI: 3.7–155.0), and frequency of replacing chicken droppings (OR = 28.3, 95% CI: 2.8–284.2). Policies addressing these risk factors will increase the effectiveness of prevention and control strategies reducing the risk of avian influenza on commercial chicken farms.

## Introduction

Chickens are the predominant species raised on commercial poultry farms in Bangladesh (1). On commercial broiler farms, chickens are reared for meat, while on commercial layer farms, chickens are raised to produce eggs, although at the end of the production cycle, spent layer hens are sold for meat (2). Broiler chickens are reared on the floor of houses (usually without solid walls), where rice husk, sawdust, and wood shavings are used as litter (3). Similar to broilers, layer chickens are also often reared in sheds without solid walls, but their management system is more complex (4). Day-old chicks layer chickens are reared on litter until grower age (pullets) and are then placed into cages where they are reared till the end of the production cycle (5). The majority of commercial farms in Bangladesh are small-scale (flock-size ≤2,000 birds) with low to minimal biosecurity (6), and only 4% of commercial farms are large-scale units rearing more than 3,000 birds with moderate to high biosecurity (1, 7).

Commercial chicken production is the main supplier of animal protein in Bangladesh, with 6.3 kg of broiler meat (8) and 103 eggs (9) consumed per capita annually. As the demand for poultry meat and eggs increases, local broiler, and layer chicken production has undergone rapid growth, resulting in a 2.5-fold increase in commercial poultry farm density between 1995 and 2017 (10, 11).

However, since 2007, the circulation of Highly Pathogenic Avian Influenza (HPAI) H5N1 and Low Pathogenic Avian Influenza (LPAI) H9N2 virus subtypes have become a major threat to chicken production in Bangladesh (12). In response to the incursion of HPAI viruses in the country, the government, with technical assistance from the World Health Organization and Food and Agriculture Organization of the United Nations (FAO), developed the first National Avian Influenza and Human Pandemic Influenza Preparedness and Response Plan (NAIPPP) for the period 2006–2008 (13). While the second NAIPPP was drafted in 2008, to cover the period 2009–2011, it has not been approved (14), leaving Bangladesh without any national policy framework to tackle the threat posed by avian influenza viruses (AIVs). The decline of reported H5N1 outbreaks in poultry since 2013, and a single human death reported to be caused by the virus means that the development and implementation of HPAI control policies are not considered a priority in Bangladesh (15). However, studies conducted in farms and live bird markets (LBMs) have shown that H5N1 and H9N2 virus subtypes circulate in Bangladeshi poultry (16, 17), with the low number of outbreaks being likely due to underreporting by farmers (12, 15). A recent study conducted on clinically affected or dead chickens with suspected AIV infection on 262 farms in Bangladesh, reported a prevalence of 4.4 and 10.1% across broiler, and 25.6 and 14.1% across layer farms, for H5 and H9 AIV subtypes, respectively (18). Interestingly, research in apparently healthy broilers and layers in Bangladesh did not detect the H5 AIV subtype, but 1.9 and 2% of the broiler and layer farms were H9 AIV positive, respectively (19).

Biosecurity is an important tool for controlling and preventing H5N1 and H9N2 dissemination in poultry populations (20). While biosecurity guidelines were developed in 2010 (21), many of the recommendations are considered impractical for small-scale farmers in Bangladesh (22).

Case-control studies conducted more than 10 years ago have highlighted biosecurity-related factors associated with an increased risk of H5N1 outbreaks in Bangladeshi chicken flocks (23–25). However, risk factors associated with the current circulation of AIVs in commercial flocks which did not report any large-scale mortalities have not been described. Furthermore, as the management of broiler and layer chicken flocks differs, there might be different pathways for H5N1 and H9N2 introduction into such farms (26, 27). Indeed, a meta-analysis (28) has highlighted that risk factors for AIV infections vary with the type of poultry production.

Therefore, to address this gap, this study aimed to identify farm-level risk factors associated with H5 and H9 infections in apparently healthy layer and broiler chickens in Bangladesh to establish more effective prevention and control strategies to reduce the risk of AIV infection in these farms.

## Materials and Methods

### Overview of the Study Design

A cross-sectional study was conducted in the Chattogram (previously, Chittagong) and Cox's Bazaar districts from February to April 2017. It involved 106 commercial broiler and 113 commercial layer chicken farms. Of the 113-layer farms, 13 had their chickens vaccinated against H5 and were, therefore, excluded from the analysis. All broiler chickens were unvaccinated against H5. Sample size calculation and farm selection have been described in detail in Gupta et al. (19). Briefly, a two-stage sampling approach (29) was used to estimate the number of farms and the number of chickens to be sampled per farm. The parameters used for sample size calculations are described in detail in Supplementary Table 1. The expected bird- and flock-level H5 antibody prevalence, i.e., the design prevalence was assumed based on Hasan (30). The chickens were randomly selected from different areas of the poultry shed without being influenced by the appearance of the chickens, such as plumage, color, and body weight and all birds sampled were clinically healthy.

### Questionnaire Design

**Figure 1:**
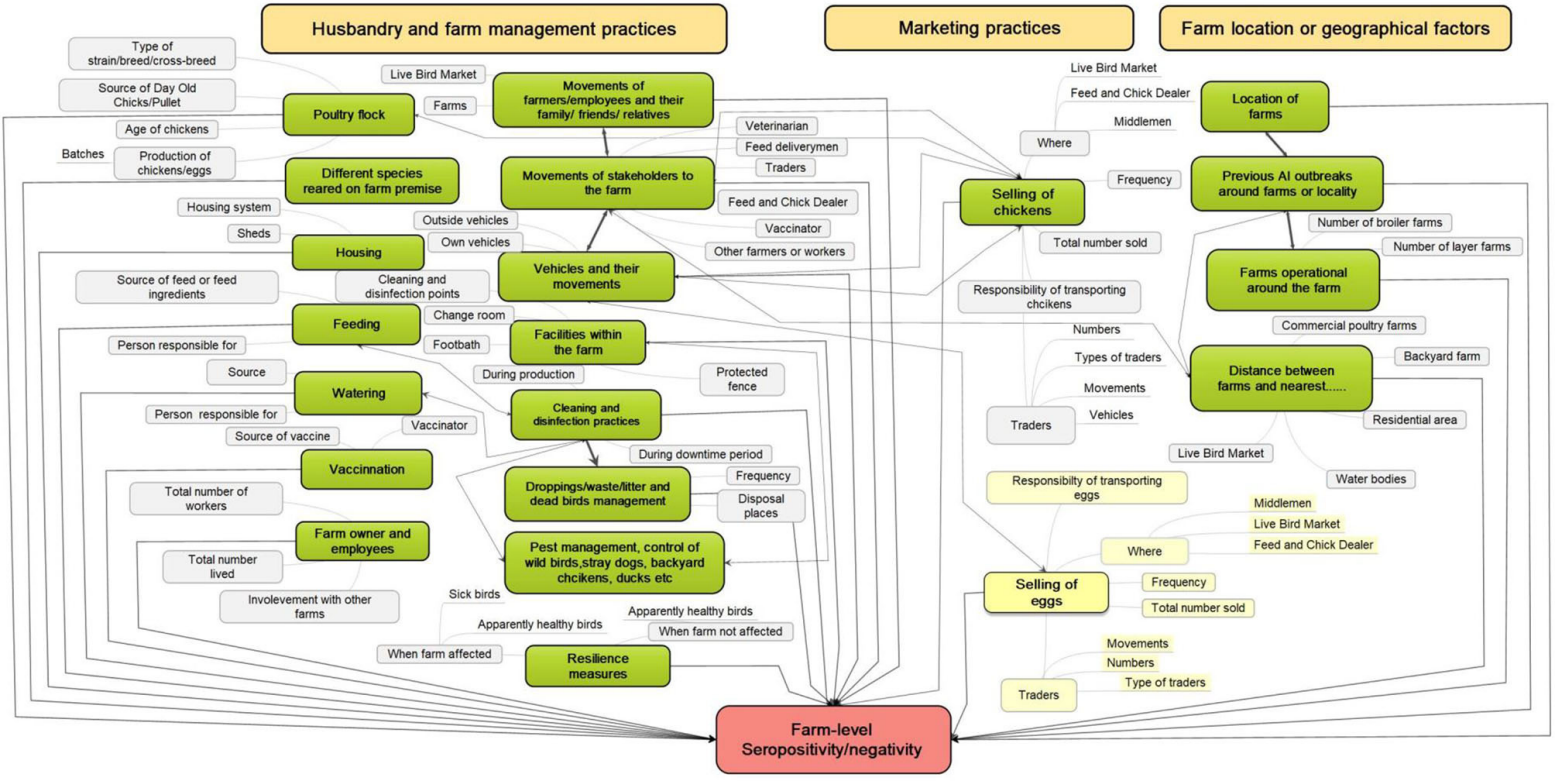
Hypothesized causal pathways for farm-level risk factors associated with avian influenza infection on the commercial broiler and layer chicken farms in Bangladesh. The red box represents the outcome (farm-level seropositivity) in the risk factor analysis, green boxes represent individual risk factors with gray boxes indicating additional categories/levels within the risk factor. The yellow boxes represent risk factors specific to layer farms. Orange boxes represent themes or categories under which risk factors can be combined. The causal pathways were used to inform the development of questions used in the interviews and to guide the inclusion of potential confounders and interactions in the final multivariable model.

Hypothetical causal pathways that could potentially increase the risk of H5 and H9 infection of broiler and layer farms (Figure 1) were developed using MindMaple Lite v1.3 (MindMaple Inc., Tustin, USA). Based on these hypothesized causal pathways, a structured questionnaire was developed in English. It focused on farmers' husbandry, management, and marketing practices, and was administered using the digital application CommCare software (Dimagi, Inc., Cambridge, USA). Although causal pathways were not used to inform the construction of multivariable statistical models in a dynamic causal framework (31), they were used to guide the inclusion of confounders and potential interactions between risks factors.

The questionnaires for broiler and layer farmers included the same 84 questions. The layer farm questionnaire also included six additional questions about the sale of eggs. The questionnaires were pilot-tested with five broilers and five-layer farmers who were not part of the study participants. After pitot-testing, minor modifications were made to nine questions.

### Data Collection

A total of 106 broilers and 100 unvaccinated layer commercial chicken farm owners (referred to as farmers) were interviewed. The interview lasted about 30 min. Prior to this, written informant consent (signature or thumb impression) was obtained. All interviews were conducted by one female and one male trained field veterinarian.

Blood samples were collected from 9 and 8 chickens on each layer and broiler farm, respectively. Depending on its body weight, 1–3 ml of blood were collected from the wing or jugular vein of a chicken and transferred into an individual sterile plastic tube immediately after collection. Tubes were kept in a cool box filled with ice packs and transported to the Chattogram Veterinary and Animal Sciences University (CVASU) laboratory (for samples collected in Chattogram) or the local District Livestock Services office (for samples collected in Cox's Bazaar). Samples were refrigerated overnight, then, the serum was separated by centrifugation at 10,000 rpm for 30 min at 4°c and transferred to Eppendorf tubes.

All sera were further processed at the CVASU laboratory. They were first screened for the presence of antibodies against Influenza A virus using commercial Enzyme-linked Immunosorbent Assay (ELISA) kits using either the IDEXX^®^ AI ELISA (product code: 5004.00, IDEXX Laboratories, Inc., USA; sensitivity 100%, specificity 99.6%) or the ID Screen^®^ Influenza A Antibody Competition Multi-Species ELISA (product Code: FLUACA ver 1216 GB, ID.vet, France; sensitivity 98.7%, specificity 98.7%).

Manufacturer recommended cut-off values were used to consider samples to be antibody positive for Influenza A. For the IDEXX^®^ AI ELISA, a sample-to-positive ratio of >0.50 was considered the cut-off value for antibody positivity, while for the ID Screen^®^ Influenza A Antibody Competition Multi-Species ELISA, a sample-to-negative ratio of ≤0.45 was used as a cut-off.

The ELISA-positive samples were then tested for the presence of H5 and H9 specific antibodies using the hemagglutination inhibition (HI) test (sensitivity 98.8%, specificity 99.5%) (32). Inactivated antigens used in the HI test were H5N1-A/Ck/Scot/59, H5N3-A/Teal/Eng/7394-2805/06, H9N2-A/Tky/Wisc/1/66, H9N9-A/knot/Eng/SV497/02 with hemagglutination (HA) titers of 2^6^, 2^7^, 2^9^, and 2^6^ for the four antigens, respectively. All antigens were produced by the Animal & Plant Health Agency, Surrey, United Kingdom. A serum sample was considered positive in the HI test when there was an inhibition at a dilution of 1/16 (2^4^) or more against 4 hemagglutinating units of antigen (33). The cut-off value of 2^4^ is recommended by OIE (33) and has been considered preferable for determining seropositivity in countries where H5N1 HPAI is endemic (34), such as in Bangladesh, where major reservoirs of H5N1 HPAI and H9N2 LPAI subtypes do exist (12, 15).

### Data Analyses

The questionnaire data were downloaded as a comma-separated values (CSV) file from the CommCare web platform and imported into STATA 14.1 (Stata Corporation, College Station, Texas, USA) for data analysis.

A causal framework of risk factors potentially being associated with avian influenza was only used to guide the development of questions for the questionnaire, but causal inference approaches were not used in the data analysis (31).

A flock (or farm) was considered seropositive for H5 (or H9), if at least one chicken was HI-positive for H5 (or H9). The analysis was conducted separately for H5 and H9, and broiler and layer farms.

A total of 344 and 421 dichotomous and ordinal variables were derived from the questionnaire data for broiler and layer chicken farms, respectively.

To reduce the number of predictors to be considered in the regression models, we used correlation analysis and screening of variables based on bivariate unconditional associations (31). As all the risk factor variables were dichotomous or ordinal, pairwise correlations were examined by estimating polychoric correlations (35) using the –*polychoric*- command in STATA. If the correlation was ≥0.9 for H5 (or H9), the biologically more plausible variable was maintained, while the other variable was removed.

Binomial logistic regression was used to assess the unconditional association between H5 (or H9) flock-level serological status and each risk factor separately. Risk factors associated with a *P*-value of ≤0.15 were included in the multivariable analysis (31).

Multivariable binominal logistic regression models were built using a backward stepwise elimination procedure. At each step, the risk factor with the highest *P*-value was removed until all factors retained in the final model had *P*-values of <0.05. For ordinal risk factors with more than two levels, we ran the Wald test using the -*testparm*- command in STATA. We also tested for confounding by subsequently adding eliminated risk factors that were biologically plausible confounders based on hypothesized causal pathways, and a ≥30% change in Odds Ratio (OR) was considered an indication of confounding (31). Biologically plausible 2-way interactions between risk factors in the final models were also explored (31).

The Hosmer-Lemeshow goodness-of-fit statistic was used to access the fit of the final model (36). Pearson and Deviance residuals and Pregibon leverage were examined to explore if any specific observations influenced the fit of the models. Finally, to evaluate the model predictive power, the area under the receiver operating characteristic (ROC) curve was calculated (36).

## Results

No HPAI outbreaks or abnormal mortality rates were reported in the 12 months preceding sampling on any of the recruited broiler (*N* = 106) and layer (*N* = 100) farms.

In the univariate analysis, we did not find any significant association between H5 (or H9) flock-level serological status and the age of chickens. However, assuming seropositivity would increase with the age of chickens and might be related to the other risk factors identified, we considered age as a potential confounder in the multivariable analysis, although did not find any indication of confounding by age.

### H5 and H9 Flock-Level Serological Status

Among the sampled broiler flocks, 9.4% (*N* = 10) and 5.7% (*N* = 6) were H5 and H9 seropositive, respectively. Similarly, in the broiler flocks, seroprevalence for H5 was higher than for H9 in layer flocks: 31% (*N* = 31) and 22% (*N* = 22), respectively.

### Farm-Level Risk Factors Associated With H5 and H9 Flock-Level Seroprevalence in Broiler Farms

Of the 344 potential risk factors examined for association with H5 and H9 seropositivity of broiler farms, nine were associated with H5 seropositivity and nine with H9 (Table 1) and were kept for the multivariable analysis. Six were identical for H5 and H9 (Table 1). Two risk factors were retained in each of the final models. Broiler farms that were visited by other farm workers during the sample production cycle were associated with higher odds of both H5 and H9 infections. Furthermore, access of ducks from neighboring backyard farms to the commercial broiler farms and an increased number of employees on the broiler farms were associated with higher odds of H5 and H9 infection, respectively (Table 1).

**Table 1:** Results of the univariate and multivariable analysis of farm-level risk factors associated with H5 and H9 flock-level seroprevalence on broiler farms in Bangladesh, 2017.

**Risk factors** **(listed in risk groups)** **(*N* = 106)**	**Category**	**Univariate analysis**								**Multivariable analysis**			
		**H5 positive (%)**	**H5 negative (%)**	**H5 OR** **(95% CI)**	**H5 *P*-Value**	**H9 positive (%)**	**H9 negative (%)**	**H9 OR** **(95% CI)**	**H9 *P*-Value**	**H5 OR** **(95% CI)**	**H5 *P*-Value**	**H9 OR** **(95% CI)**	**H9 *P*-Value**
**Access to backyard ducks**													
Access of ducks from neighboring backyard farms to the commercial farm	No	1 (1.5)	67 (98.5)	Reference	**0.005**	1 (1.5)	67 (98.5)	Reference	**0.038**	Reference	**0.007**	–	–

	Yes	9 (23.7)	29 (76.3)	20.8 (2.5–171.7)		5 (13.2)	33 (86.8)	10.2 (1.1–90.4)		21.5 (2.3–201.1)		–	
**Farm management**													
Owner involved in taking care (feeding, watering, cleaning etc.) of chickens on sampled farm	No	4 (18.2)	18 (81.8)	Reference	**0.128**	–	–	–	–	–	–	–	–
	Yes	6 (7.1)	78 (92.9)	0.3 (0.1–1.4)		–	–	–		–		–	
**Disposal of litter/waste/droppings**													
Litter/droppings/waste are disposed on commercial farm	No	7 (6.9)	94 (93.1)	Reference	**0.003**	4 (4.0)	97 (96.0)	Reference	**0.008**	–	–	–	–
	Yes	3 (60.0)	2 (40.0)	20.1 (2.9–141.2)		2 (40.0)	3 (60.0)	16.2 (2.1–125.5)		–		–	
**In- and out farm movements**													
Farm owner works or manages another commercial poultry farm	No	–	–	–	–	2 (2.6)	76 (97.4)	Reference	**0.040**	–	–	–	–
	Yes	–	–	–		4 (14.3)	24 (85.7)	6.3 (1.1–36.8)		–		–	
Workers from another commercial chicken farm visited the commercial farm during the current production cycle	No	4 (4.4)	87 (95.6)	Reference	**0.000**	1 (1.1)	90 (98.9)	Reference	**0.001**	Reference	**0.002**	Reference	**0.001**
	Yes	6 (40.0)	9 (60.0)	14.5 (3.4–61.2)		5 (33.3)	10 (66.7)	45.0 (4.8–424.5)		15.1 (2.8–80.8)		50.1 (4.5–552.7)	
Private veterinarians visited the commercial farm in the current production cycle	No	8 (13.6)	51 (86.4)	Reference	**0.122**	–	–	–	–	–	–	–	–
	Yes	2 (4.3)	45 (95.7)	0.3 (0.1–1.4)		–	–	–		–		–	
Total number of vehicles (rickshaw van, pick-up, motorized vehicle etc.) used by traders to collect the last batch of chickens on the commercial farm	0 to 5	4 (6.1)	62 (93.9)	Reference	**0.139**	2 (3.0)	64 (97.0)	Reference	**0.150**	–	–	–	–
	>5	6 (15.0)	34 (85.0)	2.7 (0.7–10.4)		4 (10.0)	36 (90.0)	3.6 (0.6–20.4)		–		–	
Total number of workers on the commercial farm	0 to 1	5 (6.1)	77 (93.9)	Reference	**0.040**	2 (2.4)	80 (97.6)	Reference	**0.021**	–	–	Reference	0.041
	≥2	5 (20.8)	19 (79.2)	4.1 (1.1–15.4)		4 (16.7)	20 (83.3)	8.0 (1.4–46.8)		–		9.4 (1.1–80.6)	
**Marketing practices**													
Sale of the last batch of broiler chickens to a Feed and Chick Dealer (FCD)	No	2 (4.1)	47 (95.9)	Reference	**0.100**	–	–	–	–	–	–	–	–
	Yes	8 (14.0)	49 (86.0)	3.8 (0.8–19.0)		–	–	–		–		–	
**Farm characteristics**													
Total number of sheds on the commercial farm	1 to 2	–	–	–	–	3 (3.5)	84 (96.6)	Reference	**0.054**	–	–	–	–
	3 to 4	–	–	–		3 (15.8)	16 (84.2)	5.3 (1.0–28.4)		–		–	
**History of AI outbreaks near farm**													
AI outbreaks near the commercial farm or within the village within the last 12 months	No	7 (7.1)	91 (92.9)	Reference	**0.013**	4 (4.1)	94 (95.9)	Reference	**0.033**	–	–	–	–
	Yes	3 (37.5)	5 (62.5)	7.8 (1.5–39.6)		2 (25.0)	6 (75.0)	7.8 (1.2–51.7)		–		–	
**Farm location or geographical factors**													
Total number of broiler farms operating within 0.5 km of the commercial farm	0–2	–	–	–	–	1 (1.6)	61 (98.4)	Reference	**0.065**	–	–	–	–
	≥3	–	–	–		5 (11.4)	39 (88.6)	7.8 (0.9–69.5)		–		–	

*Values in bold in the univariate analysis represent risk factors associated with a P-value of ≤ 0.15 that were included in the multivariable analysis. The Values in bold in the multivariable analysis represent risk factors associated with a P-value of <0.05 that were retained in the final model*.

### Farm-Level Risk Factors Associated With H5 and H9 Flock-Level Seroprevalence in Layer Farms

Of the 421 potential risk factors associated with H5 and H9 seropositivity on layer farms, 13 risk factors for H5 and 11 for H9 seropositivity were kept for the multivariable analysis (Table 2). Three were identical for H5 and H9 (Table 2). Four and five risk factors were retained for the H5 and H9 final models, respectively (Table 2). The presence of stray dogs on the farm was associated with higher odds of both H5 and H9 infections. Also, allowing outside vehicles to enter the farms (to deliver feed, DOCs, or to collect litter and droppings), a larger number of workers on the farms, and the burying of dead birds near the commercial farms were associated with increased odds of H5 infection.

**Table 2:** Results of the univariate and multivariable analysis for farm-level risk factors associated with H5 and H9 flock–level seroprevalence on layer farms in Bangladesh, 2017.

**Risk factors** **(listed in risk groups)** **(*N* = 100)**	**Category**	**Univariate analysis**								**Multivariable analysis**			
		**H5 positive (%)**	**H5 negative** **(%)**	**H5 OR (95% CI)**	**H5 *P*-Value**	**H9 positive (%)**	**H9 negative (%)**	**H9 OR (95% CI)**	**H9 *P*-Value**	**H5 OR (95% CI)**	**H5 *P*-Value**	**H9 OR** **(95% CI)**	**H9 *P*-Value**
**Source of the DOC/pullets and feed**													
DOC or pullets were obtained from a hatchery or breeding farm	No	30 (35.7)	54 (64.3)	Reference	**0.045**	–	–	–	–	Reference	**0.003**	–	–
	Yes	1 (6.3)	15 (93.8)	0.1 (0.0–1.0)		–	–	–		0.0 (0.0–0.3)		–	
Feed and Chick Dealer (FCD) provided feed or feed ingredients	No	–	–	–	–	2 (7.4)	25 (92.6)	Reference	**0.047**	–	–	Reference	**0.049**
	Yes	–	–	–		20 (27.4)	53 (72.6)	4.7 (1.0–21.8)		–		5.9 (1.0–33.9)	
**Stray dogs**													
Access of stray dogs to the commercial farm	No	13 (24.5)	40 (75.5)	Reference	**0.140**	8 (15.1)	45 (84.9)	Reference	**0.081**	Reference	**0.040**	Reference	**0.039**
	Yes	18 (38.3)	29 (61.7)	1.9 (0.8–4.5)		14 (29.8)	33 (70.2)	2.4 (0.9–6.3)		3.1 (1.1–9.1)		4.0 (1.1–15.3)	
**In- and out farm movements**													
Farm owner worked or managed another commercial poultry farm	No	–	–	–	–	14 (17.7)	65 (82.3)	Reference	**0.051**	–	–	–	–
	Yes	–	–	–		8 (38.1)	13 (61.9)	2.9 (1.0–8.2)		–		–	
Visits of LBMs in the last month by farmers, workers or family members that had access to the commercial farm	No	–	–	–	–	8 (15.7)	43 (84.3)	Reference	**0.124**	–	–	–	–
	Yes	–	–	–		14 (28.6)	35 (71.4)	2.2 (0.8–5.7)		–		–	
Frequency of LBM visits in the last month by farmers, workers or family members that had access to the commercial farm	0 times	14 (27.5)	37 (72.6)	Reference	**0.027**	–	–	–	–	–	–	–	–
	1 to 10 times	2 (11.1)	16 (88.9)	0.3 (0.1–1.6)		–	–	–		–		–	
	>10 times	15 (48.4)	16 (51.6)	2.5 (1.0–6.3)		–	–	–		–		–	
Visits of other commercial poultry farms in the last month by farmers, workers or family members who had access to the commercial farm	No	–	–	–	–	16 (18.8)	69 (81.2)	Reference	**0.076**	–	–	Reference	**0.039**
	Yes	–	–	–		6 (40.0)	9 (60.0)	2.9 (0.9–9.2)		–		4.7 (1.1–20.6)	
Feed delivery on commercial farm in the current production cycle	No	20 (26.7)	55 (73.3)	Reference	**0.109**	–	–	–	–	–	–	–	–
	Yes	11 (44.0)	14 (56.0)	2.2 (0.8–5.5)		–	–	–		–		–	
Commercial farm used its own vehicle for farm activities/movements	Yes	5 (17.2)	24 (82.8)	Reference	**0.064**	3 (10.3)	26 (89.7)	Reference	**0.084**	–	–	–	–
	No	26 (36.6)	45 (63.4)	2.8 (0.9–8.1)		19 (26.8)	52 (73.2)	3.2 (0.9–11.7)		–		–	
Vehicles entered the commercial farm (excluding vehicles of traders who purchased chicken or eggs)	No	4 (15.4)	22 (84.6)	Reference	**0.053**	–	–	–	–	Reference	**0.011**	–	–
	Yes	27 (36.5)	47 (63.5)	3.2 (1.0–10.1)		–	–	–		5.8 (1.5–22.4)		–	
Total number of workers on the commercial farm	0 to 2	13 (22.0)	46 (78.0)	Reference	**0.062**	–	–	–	–	Reference	**0.013**	–	–
	3 to 4	11 (40.7)	16 (59.3)	2.4 (0.9–6.5)		–	–	–		4.8 (1.4–16.3)		–	
	≥5	7 (50.0)	7 (50.0)	3.5 (1.0–11.9)		–	–	–		5.8 (1.2–28.2)		–	
**Marketing practices**													
Total number of spent layers sold in the last batch from the commercial farm	0 to ≤ 950	–	–	–	–	7 (13.0)	47 (87.0)	Reference	**0.044**	–	–	Reference	**0.004**
	>950 to ≤ 2,000	–	–	–		7 (26.9)	19 (73.1)	2.5 (0.8–8.0)		–		5.9 (1.2–29.1)	
	>2,000	–	–	–		8 (40.0)	12 (60.0)	4.5 (1.4–14.8)		–		24.0 (3.7–155.0)	
Frequency of sales of spent layers sold from the last batch	0 to 1 time	15 (23.1)	50 (76.9)	Reference	**0.022**	–	–	–	–	–	–	–	–
	≥2 times	16 (45.7)	19 (54.3)	2.8 (1.2–6.8)		–	–	–		–		–	
Sale of the last batch of spent layers to a Feed and Chick Dealer (FCD)	No	23 (26.7)	63 (73.3)	Reference	**0.029**	16 (18.6)	70 (81.4)	Reference	**0.050**	–	–	–	–
	Yes	8 (57.1)	6 (42.9)	3.7 (1.1–11.7)		6 (42.9)	8 (57.1)	3.3 (1.0–10.8)		–		–	
Minimum number of spent layers sold over the last 24 months	0 to <1,700	17 (25.0)	51 (75.0)	Reference	**0.113**	–	–	–	–	–	–	–	–
	≥1,700 to ≤ 2,000	7 (53.9)	6 (46.2)	3.5 (1.0–11.9)		–	–	–		–		–	
	>2,000	7 (36.8)	12 (63.2)	1.8 (0.6–5.2)		–	–	–		–		–	
Minimum number of eggs sold per sale in the last month	0 to 1,000	–	–	–	–	4 (11.1)	32 (88.9)	Reference	**0.080**	–	–	–	–
	1,001 to 5,000	–	–	–		12 (24.5)	37 (75.5)	2.6 (0.8–8.8)		–		–	
	>5,000	–	–	–		6 (40.0)	9 (60.0)	5.3 (1.2–23.1)		–		–	
**Cleaning practices and disposal of dead birds**													
Frequency of replacing litter or droppings during the current production cycle on the commercial farm	Daily or weekly	–	–	–	–	14 (17.5)	66 (82.5)	Reference	**0.060**	–	–	Reference	**0.013**
	Fortnightly, monthly or >monthly	–	–	–		4 (30.8)	9 (69.2)	2.1 (0.6–7.8)		–		4.6 (0.7– 29.0)	
	Not at all	–	–	–		4 (57.1)	3 (42.9)	6.3 (1.3–31.3)		–		28.3 (2.8– 284.2)	
Sale of litter or droppings to fish farmers	No	27 (35.1)	50 (64.9)	Reference	**0.116**	–	–	–	–	–	–	–	–
	Yes	4 (17.4)	19 (82.6)	0.4 (0.1–1.3)		–	–	–		–		–	
Burying of dead birds near the commercial farm	No	4 (14.8)	23 (85.2)	Reference	**0.040**	–	–	–	–	Reference	**0.026**	–	–
	Yes	27 (37.0)	46 (63.0)	3.4 (1.1–10.8)		–	–	–		4.6 (1.2–17.3)		–	
Garbage piled up near the chicken sheds on the commercial farm	No	–	–	–	–	6 (14.3)	36 (85.7)	Reference	**0.119**	–	–	–	–
	Yes	–	–	–		16 (27.6)	42 (72.4)	2.3 (0.8–6.5)		–		–	
**Farm location or geographical factors**													
Total number of layer farms	0	13 (23.6)	42 (76.4)	Reference	**0.066**	–	–	–	–	–	–	–	–
operating within 0.5 km of the	1	10 (32.3)	21 (67.7)	1.5 (0.6–4.1)		–	–	–		–		–	
commercial farm	>1	8 (57.1)	6 (42.9)	4.3 (1.3–14.7)		–	–	–		–		–	

*LBM, Live Bird Market; DOC, Day Old Chick. Values in bold in the univariate analysis represent risk factors associated with a P-value of ≤ 0.15 that were included in the multivariable analysis. The Values in bold in the multivariable analysis represent risk factors associated with a P-value of <0.05 that were retained in the final model*.

Layer farms, which were supplied with feed or feed ingredients through FCDs, layer farms of which farmers, workers, or their family members visited other commercial poultry farms and layer farms from which a large number of spent layers were sold in the last batch and farms with limited replacement of litter or droppings were associated with higher odds of H9 seropositivity. In contrast, layer farms that were supplied directly by hatchery or breeder farms with DOC or pullets had lower odds of H5 infection.

For all multivariable models, the Hosmer–Lemeshow goodness-of-fit statistics were associated with a *P*-value of >0.37, indicating a good fit. The Area Under the ROC Curve was always at >0.82, indicating good predictive power and the ability of the four models to discriminate between seropositive and seronegative farms (36).

## Discussion

This is the first research study in an H5N1-endemic country that identified risk factors associated with H5 and H9 seropositivity in clinical healthy commercial broilers and layers. In fact, in the 12 months preceding the sampling of birds, no major mortalities or clinical symptoms suggestive of HPAI infection were observed on any of these commercial chicken farms. None of the birds were vaccinated against H5 AIV infection, although some birds did develop antibodies. One possible explanation for the detection of these H5 antibodies (with no occurrence of HPAI clinical signs or mortalities) might be the infection with LPAI H5 strains. In Bangladesh, LPAI H5N2 was reported by Gerloff et al. (37), and in Asia, other LPAI H5 viruses (H5N3, H5N8) including H5N2 were described by Duan et al. (38) and Nguyen et al. (39). It could also be possible that endemicity of H5 AIV in Bangladesh might have resulted in reduced pathogenicity due to viral evolution (40) or that H5 AIV infected birds show milder disease symptoms due to the development of cell-mediated immunity contributing to host resistance (41).

We identified that the presence of ducks raised on neighboring backyard farms in the recruited broiler farms increased the odds of H5 seropositivity. Free-grazing ducks have been reported to be associated with HPAI outbreak occurrence in Thailand in 2004 (42). In Bangladesh, many backyard farmers rear ducks along with chickens (43). Domestic ducks are usually left to scavenge around village households, on ponds and wetlands, or other agricultural lands (44) and might enter commercial poultry farms. While it is unlikely that roaming ducks can enter chicken broiler sheds, duck droppings might contaminate the farm environment, and workers (*via* their clothes or shoes, etc.) or farm equipment (e.g., waterer and feeders) act as mechanical vectors exposing broiler chickens to the virus.

Visits by workers from other commercial chicken farms increased the odds of both H5 and H9 seropositivity. This underpins the importance of human movements for H5 and H9 disease spread (45, 46). The odds of H9 seropositivity on broiler farms were also increased if the number of employees on the farm was high. More employees will result to more movements and potentially contaminating contacts. A case-control study conducted in Bangladesh also identified the number of employees as a risk factor for H5N1 outbreak occurrence on commercial chicken farms (25).

The presence of stray dogs was associated with increased odds of H5 and H9 seropositivity on layer farms. A previous case-control risk factor study conducted in Bangladesh highlighted that the presence of feral or wild animals, including dogs, was associated with H5N1 infection on commercial chicken farms (23). Surveillance data of canine populations in southern China reported high rates of antibody positivity (44.85%) and isolation of avian H9N2 virus from some dogs (47). It has been shown experimentally that the H9N2 virus isolated from broiler chickens was able to infect dogs, which were consequently able to shed the virus (48). In Thailand, the death of one dog following the ingestion of an H5N1-infected duck has been reported (49). Thus, dogs that become sub-clinically infected with the H5N1 virus could contribute to the spread of the virus, and it is recommended that dog-poultry contact should be avoided to mitigate the potential spread of the virus (50).

In Bangladesh, the high number of stakeholders involved in poultry production can promote the spread of H5N1 (20, 51). The purchase of day-old chicks (DOCs) or pullets directly from hatcheries or breeding farms reduced the risk of H5 seropositivity in layer chickens compared to their purchase from feed and chick dealers (FCDs) or through middlemen. FCDs supply DOC, feed, medicine, and equipment to commercial farms. They also regularly visit farms to provide advice on disease management, and might be in contact with sick or dead birds. Hatcheries, on the other hand, only produce chicks. Having high biosecurity standards, they may be less likely to be a source of infection for farms. Similarly, the involvement of FCDs in the supply of feed or feed ingredients increased the odds of H9 seropositivity.

The disposal of carcasses can be a challenge for commercial chicken farmers (52). Farmers burying dead birds near their farm premises had higher odds of H5 seropositivity. Poultry carcasses may be disposed of through burial, incineration, composting, and rendering (53). However, some countries have banned the burial of dead birds due to the rise of groundwater being contaminated by pathogens (52). Nevertheless, if the burial of dead birds is conducted, carcasses need to be buried deeply so that feral and wild animals are not able to retrieve carcasses (54). In Bangladesh, carcasses that are not appropriately disposed may attract dogs, jackals, and foxes (22, 55). Yet, as HPAI viruses may remain infectious in carcasses for up to 6 days at 22–23°C (56), such carcasses from infected birds may become a source of infection.

Low frequency to unchanging litter or cleaning droppings in chicken houses during the production cycle was also associated with increased odds of H9 seropositivity in layer chickens. Kurmi et al. (57) estimated that AIV can survive 5 days at 24°C and 8 weeks at 4°C in dry and wet feces, respectively, while survival of AIV in poultry sheds for up to 5 weeks had been reported by others (58). Thus, poultry litter can provide a favorable environment for AIV spread.

Farms in which vehicles could enter to deliver feed, DOCs, or to collect litter, droppings had increased odds of H5 seropositivity. Vehicles moving between farms may be able to spread AIV (59). For instance, poultry droppings are used as feed by fish farmers in Bangladesh (60) and are usually collected from multiple poultry farms.

Layer farms in which farmers, workers, or their family members visited other commercial poultry farms had higher odds of H9 infection. It has been linked to an increased risk of HPAI outbreaks in another study (61).

Odds of H9 seropositivity increased with the number of spent layers (>2,000) sold from the last batch. Sales of a larger number of birds might involve a larger number of traders or middlemen visiting the farm premises. In Hong Kong (46), the visits to farms by more than one person from retail markets were found to be a risk factor for H5N1 infection.

This study had some limitations. Firstly, some of the information collected on chicken marketing and production referred to the last 12 months and, therefore, relied on recall by farmers. However, we tried to limit recall bias by simplifying the questions, and focusing on dichotomized or simple ordinal responses. Secondly, despite the inclusion of questions about seasonal changes in sales and flock size, our investigation of the impact of seasonal factors on H5 and H9 seasonality was limited by the cross-sectional nature of the study.

The risks of AIV infections on broiler and layer farms could be mitigated through appropriate management of the risk factors identified in this study. Roaming of scavenging ducks and stray dogs should be prevented and the erection of protective fences around commercial farms is highly advisable. Workers (or their family members) from other commercial poultry farms and traders or middlemen should not be allowed to enter chicken sheds or houses without the implementation of precautionary measures. If it is not feasible to restrict the movements of farmers, workers, or traders, hand and foot washing facilites, as well as rooms for changing footwear and clothes, should be set up. In addition, vehicles need to be properly cleaned and disinfected before entering and leaving farm premises and, if possible, should not be parked within 30 meters of chicken sheds (21). DOCs, pullets, and poultry feed should be purchased from reliable sources with good biosecurity, while daily, or at least weekly, cleaning of litter and deep burial of dead birds as far as possible from the farms, will also reduce the risk of H5 and H9 spread.

Unfortunately, the implementation of AI prevention and control measures is strongly influenced by farmers' perceptions about the measures to be introduced (e.g., wearing protective equipment might be considered by farmers as an impediment to effectively handling chickens) (62). In addition, limited financial resources and the actual cost of interventions might also constrain the implementation of improved biosecurity on farms (63, 64).

Therefore, farmers need to be educated in risk-reducing behaviors, how to choose production input suppliers, and how biosecurity can be improved, without large financial burdens. Thus, the findings of this study will help policymakers to develop more effective prevention and control strategies to reduce the risk of H5 and H9 infections on the commercial broiler and layer chicken farms.

## Data Availability Statement

The raw data supporting the conclusions of this article will be made available by the authors, without undue reservation.

## Ethics Statement

The studies involving human participants were reviewed and approved by Behavioural and Social Sciences Ethical Review Committee, the University of Queensland (Approval Number: 2015001703). The farmers/participants provided their written informed consent to participate in this study. The animal study was reviewed and approved by Animal Ethics Committee, Animal Welfare Unit, the University of Queensland Research and Innovation (Approval Number: SVS/465/15/RVC). Written informed consent was obtained from the owners for the participation of their animals in this study.

## Author Contributions

JH, GF, and MH obtained the funding for the field research. SG, JH, GF, and MH conceived and designed the study. The questionnaire was developed by SG with inputs from JH, GF, and MH. Data collection was conducted by SG under the supervision of MH. SG conducted data analysis under the guidance of JH. All authors offered advice on the interpretation of data. SG drafted the initial manuscript. All authors read, reviewed, revised, and approved the final version of the manuscript.

## Funding

This work was supported by the BALZAC research program Behavioural adaptations in live poultry trading and farming systems and zoonoses control in Bangladesh [grant number BB/L018993/1] and is 1 of 11 programs supported by the Zoonoses and Emerging Livestock Systems, a joint research initiative between the Biotechnology and Biological Sciences Research Council; the Defence Science and Technology Laboratory; the Department for International Development; the Economic and Social Sciences Research Council; the Medical Research Council and the Natural Environment Research Council. The first author of this publication was supported by the Australian Government through an Australian Government Research Training Program Scholarship.

## Conflict of Interest

The authors declare that the research was conducted in the absence of any commercial or financial relationships that could be construed as a potential conflict of interest.

## Publisher's Note

All claims expressed in this article are solely those of the authors and do not necessarily represent those of their affiliated organizations, or those of the publisher, the editors and the reviewers. Any product that may be evaluated in this article, or claim that may be made by its manufacturer, is not guaranteed or endorsed by the publisher.
