# Supplementary material for: Farm-Level Risk Factors Associated With Avian Influenza A (H5) and A (H9) Flock-Level Seroprevalence on Commercial Broiler and Layer Chicken Farms in Bangladesh
Source: Front Vet Sci. 2022 Jun 16;9:893721. doi: 10.3389/fvets.2022.893721 (PMC9255630; doi:10.3389/fvets.2022.893721)
Supplement: Supplementary file 1 [file Table_1.DOCX]

**Supplementary Materials**

**Table S1**. Input parameters used for two-stage sampling approach to calculate the number of farms and the number of chickens per farm to be sampled.

| Parameters | Commercial broiler chickens | Commercial layer chickens |
| --- | --- | --- |
| **Input parameters for sample size calculations** | | |
| Haemagglutination Inhibition test sensitivity (%) | 98 | 98 |
| Confidence level (%) | 95 | 95 |
| Design bird-level H5 antibody prevalence (%) | 15.0 | 35.0 |
| Design flock-level H5 antibody prevalence (%) | 25.0 | 45.0 |
| Flock size | 1500 | 1500 |
| Tolerance (%) | 10 | 10 |
| Minimum desired flock sensitivity (%) | 75.0 | 95.0 |
| Calculated flock sensitivity (%) | 76.2 | 96.6 |
| **Estimated sample size** | | |
| Number of farms to be sampled | 103 | 102 |
| Number of chickens to be sampled per farm | 9 | 8 |
